# Physical inactivity, and its association with hypertension among employees in the district of Colombo

**DOI:** 10.1186/s12889-021-12013-y

**Published:** 2021-11-29

**Authors:** A. U. Gamage, R. de A. Seneviratne

**Affiliations:** 1 Present Address: Senior Lecturer in Community Medicine, Paraclinical Department, Faculty of Medicine, General Sir John Kotelawala Defence University, Ratmalana, 10390 Sri Lanka; 2 Senior Professor in Community Medicine, Paraclinical Department, Faculty of Medicine, General Sir John Kotelawala Defence University, Ratmalana, 10390 Sri Lanka

**Keywords:** Physical-inactivity, Commuting-distance, Hypertension, Socioeconomic variations, Employees

## Abstract

**Background:**

Physical inactivity is a leading cause of morbidity and mortality and is a major public health problem. Insufficient activity is responsible for a large proportion of non-communicable diseases such as hypertension.

**Objectives:**

The purpose of this study was to assess socioeconomic variations in physical activity and to measure the association between physical inactivity and hypertension among government officials in Sri Lanka.

**Methods:**

A cross-sectional study was carried out among 275 senior-officers(SOs) and 760 managerial-assistants(MAs) aged 30–60 years and attached to Public Administration institutions in Colombo District in Sri Lanka. Physical-activity(PA) was gathered using the International Physical Activity Questionnaire(IPAQ) adopted and validated to the Sri Lankan context. Blood pressure(measured and classified using JNC-7 guidelines) and anthropometric indices were recorded. Energy utilization of all vigorous and moderated PA and walking was expressed as metabolic-equivalent-of-task(MET) min per week. A total-MET-score was calculated and categorized based on IPAQ guidelines.

**Results:**

Socioeconomic variations in PA levels were observed as 58.1%(*n* = 158) SOs and 30.6%(*n* = 226) MAs were involved in inadequate PA. Among the SOs diagnosed with hypertension, more half(59.1%; *n* = 52) were physically inactive, while among MAs, 65.9%(*n* = 143) with hypertension were physically inactive. After adjusting for potential confounding factors being physically inactive was associated with a higher risk of hypertension among SOs[OR 2.08 [95% CI 1.07, 4.6] and MAs[OR 2.8 [95% CI 1.8, 4.6]. The main modality of commuting to work for SOs(59%) was private transport, and MAs(64%) public transport Commuting distance was positively correlated(*p* < 0.05) with total transport MET among SOs and MAs. After adjusting for confounders, commuting distance of > 20 km was found to lower the odds of hypertension among SOs and MAs(OR = 0.713; 95% CI 0.4 to1.3; and OR = 0.63; 95% CI 0.46 to 0.87).

**Conclusion:**

Despite the current knowledge that being physically active promotes health, the practice was different. Physical inactivity was associated with hypertension and prevalent among both SOs and MAs. Higher commuting distance is positively correlated with total transport MET and associated with lower odds of hypertension among SOs and MAs. Longitudinal studies are required to provide a causative association between physical inactivity and hypertension among these employees.

## Introduction

Physical inactivity is a leading cause of death and, therefore, a major public health problem [1]. There is compelling evidence that physical inactivity is responsible for a large proportion of coronary heart disease and type II diabetes, and hypertension [2]. Physical inactivity led to 9% of premature deaths (5·3 million deaths) in 2008 [3, 4]. Additionally, there are socioeconomic variations observed in physical inactivity [5]. The recently published WHO recommendations on physical activity state that all adults (18- 64 years) should engage in 150–300 min of moderate-intensity, or 75–150 min of vigorous-intensity physical activity, or an equivalent combination of moderate- and vigorous-intensity activity throughout the week [6, 7]. There is epidemiological evidence that suggests a dose-dependent relationship between physical activity and hypertension [8].

Social determinants of health (SDH) are non-medical factors that influence health-related outcomes and can be altered through informed science [9]. According to WHO, the SDH are the conditions in which people are born, grow, live and work, through which complex pathways act and health-related outcomes are shaped. The SDH consists of various factors and includes education, income, working life conditions, socioeconomic position/ status/ gradient, public policies, health services, access, transport, and built environment [9, 10]. The SDHs account for 30–55% of the health outcomes and are responsible for health inequalities. The lower socioeconomic position leads to lower health outcomes [9]. Socioeconomic position/status refers to an individual’s or a group’s location in society’s social structure and is influenced by social and economic factors [11]. The social position/ status, defined by socioeconomic factors, can be proxied using various indicators. One such indicator is the job category which reflects the occupational hierarchy within a specific working population and is used in the Whitehall studies. Evidence suggests a strong and consistent relationship between these socioeconomic factors, such as job category, and physical health outcomes such as NCDs{Lago - Peñas, 2020 #1175} [10]. Moreover, socioeconomic position/status is also associated with NCD risk factors such as physical inactivity [10]. For example, evidence suggests that White-collar workers are 84% more likely to be physically inactive [12].

When assessing physical activity (PA), there are four main dimensions that are of interest; the type of, the frequency, the duration, and the intensity of the PA [13]. The type of PA is referred to the different types of activities the subjects are engaged in. The frequency of PA activity refers to the number of sessions of physical activity per unit of time. The duration is the length of time spent in each activity session. Additionally, there are four main types of daily physical activity: occupation-related, transport-related, household-related, and leisure time-related [13, 14]. The occupational activity involves activity at work and commuting to work. An occupational activity represents the greatest portion of daily time for most adults who are employed before retirement. Commuting to work is defined as regular travel between one’s residence and place of work [15]. It is believed that sedentary behavior associated with long-distance commuting hinders physical activity and ill health [15].

The development of questionnaire-based methods for assessing PA has paved the way to assess PA’s amount and patterns and determine the relationship between PA and health-related outcomes. The International Physical Activity Questionnaire (IPAQ) was developed in Geneva in 1998 [13, 14]. This is a widely used questionnaire in assessing physical activity in adults [16, 17]. Two IPAQ versions are available (a long version and a short version), and both versions are available in self-administered or interviewer-administered forms. The reliability and validity of the IPAQ were established across 12 countries in 2000, including Sri Lanka [14]. The IPAQ assesses the PA in the last seven days. The compendium was developed based on adult activities and can correctly estimate the employed population’s physical activities [14]. IPAQ assesses the energy expenditure of adults using a compendium of physical activities to estimate energy expenditure.

Metabolic equivalent of task (MET) expresses the energy expenditure (or calories) of physical activities. The total volume of physical activity can be quantified in MET-hours per day per week [18, 19]. This is the total of all different activities performed during the assessment period expressed in MET equivalent and multiplied by all time spent in all activities. Considering the MET values, the type, and the frequency of the PA, the total volume can be categorized into insufficient/low, sufficient/ moderate, or vigorous/highly activity [18, 19].

Socioeconomic variations in health conditions and related risk factors can vary between different population groups [20]. Global Action Plan on Physical activity 2018–2030 adopted a new global target to reduce physical inactivity globally by 15% by 2030 [6]. To achieve this, it is essential to assess and be aware of the disparities of health-related conditions in different groups to guide policy decisions. Given the importance of understanding variations, this study aimed to assess socioeconomic variations in physical activity and measure the association between physical inactivity and hypertension among government officials in Sri Lanka. By exploring the socioeconomic variations in physical inactivity among the study population, we aim to fill the knowledge gap related to variations in physical inactivity between SOs and MAs, enabling policymakers and healthcare providers to make better evidence-informed decisions and develop an equitable delivery of interventions, especially at the workplace.

## Methods

### Participants

A descriptive cross-sectional study was conducted among senior officers and managerial assistants attached to public administration offices in the Colombo District [21]. In this district, there are 23 such offices. The total number of senior officers (SOs) and managerial assistants (MAs) attached to the institutions were 358 and 1231, respectively. The study population comprising full-time, permanent SOs and MAs between the ages of 30 to 60 years and employed for at least one year or more in a similar government institution in a similar cadre post were selected for the study. Officers on maternity or other extended leave and officers on prolonged (more than one month) steroid therapy confirmed by documented evidence were excluded from the study [21].

The senior officers and managerial assistants were studied separately for the prevalence of hypertension and related risk factors. They are two distinct categories socioeconomically and are deemed to have a different distribution of determinants. Senior officers and managerial assistants attached to government Public Administrative offices are authorized officers to conducted administrative tasks in the country. These two populations have different job roles and responsibilities, exposing them to different determinants of ill health and related risk factors. Therefore, sample Sample size calculation was done separately for SOs and MAs.

Sample size calculation to detect the prevalence of hypertension was done assuming the prevalence of hypertension among adults was 20, 95% confidence interval, and a precision of 0.05 using the formula to detect a population proportion. Ten percent was added to account for nonresponse.

**Fig. 1:**
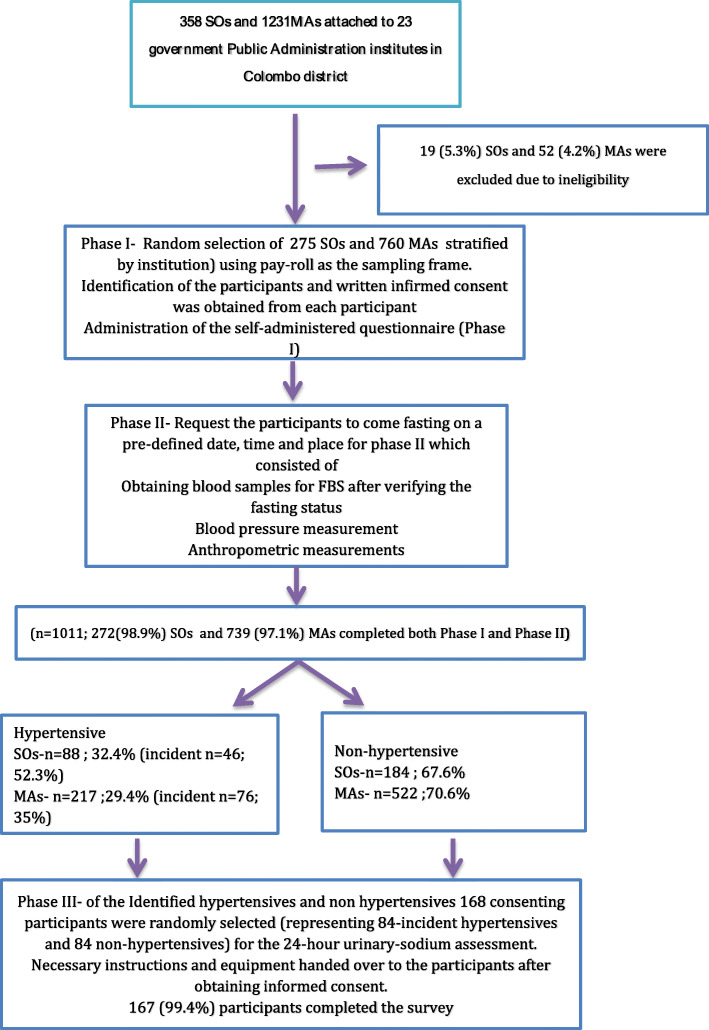
Flow of individuals through the study. Source: [22]

Thus, the final calculated sample size for SOs and MAs was 275 and 760, respectively. A stratified simple random sampling technique was used to select the SOs and MAs. Stratification was done according to the 23 institutions they are attached to, and the number needed to select from each institution was decided according to probability proportionate to the size (PPS). The required number of SOs and MAs from each institution was selected randomly based on the number allocated to each institution according to PPS. The latest updated version of the payroll was used as the sampling frame for sampling, and the completeness was checked with an institutional name list before use. A unique ID number was given to all the eligible officers. Computer-generated random numbers were used to identify the study participants. Finally, the identified officers were met face to face and invited to participate in the study. The study population recruited, sampling procedure, and phases of the study are given in Fig. 1 [22].

Ethical clearance was obtained from the Ethical Review Board of Faculty of Medicine, University of Colombo. Permission and consent were obtained from the Ministry of Public Administration and Home Affairs and all heads of institutions before the commencement of the study. Written informed consent was obtained from all respondents after informing the following in the information sheet: the purpose, the objectives, and benefits to the occupational group by conducting this study.

A self-administered questionnaire (SAQ) was used to gather information, which consisted of 3 broad components: sociodemographic characteristics; work-related information including commuting distance, which included validated IPAQ; and lifestyle-related correlates of hypertension. In addition to the SAQ, a data collection form was used to record blood pressure (BP), anthropometric measurements, fasting blood sugar values, and record information on the participant’s past medical history and drug history. To assess test-retest reliability, 10% of the questionnaires were readministered to randomly selected study participants 2 weeks after the initial data collection.

### Measures

#### Blood pressure

The BP measurements were done based on the American Heart Association BP measurement recommendations, which reduced the intra-observer error of BP measurements [23, 24]. The principal investigator carried out all BP measurements. Participants were allowed to sit for 5 min before measuring BP. The participants were asked to refrain from smoking or ingesting caffeine during the 30 min preceding the BP measurement. A cuff with a bladder that is 12–13 cm’ 35 cm in size with a larger bladder for fat arms was used. The bladder within the cuff will encircle at least 80% of the arm. The cuff was placed at the heart level of the patient. The disappearance of Phase V Korotkoff sounds was used to measure the diastolic BP. Two BP readings were obtained, separated by 1 min. The average of these two values was taken.

An additional reading was taken and averaged when the first two readings differed by more than 5 mmHg. Classification of hypertension was done based on the classification of the Joint National Committee on Prevention, Detection, Evaluation, and Treatment of High Blood Pressure (JNC-7) [25]. A person was considered hypertensive if he/she was an already diagnosed case of hypertension and/or on treatment or with a current systolic BP of ≥140 mmHg or diastolic BP ≥90 mmHg (JNC-7 criteria) [25].

#### Physical activity

The level of physical activity was assessed using the long version of the International Physical Activity Questionnaire (IPAQ), which was validated in the local setting [26]. Participants were instructed to recall and record activities they were engaged in the last seven days and record only the physical activity that lasted for at least 10 min. The long version of the IPAQ consisted of different domains such as work-related, transport-related house work-related, and sports and leisure time-related physical activity. Energy utilization of all vigorous and moderated PA and walking was expressed as metabolic-equivalent-of-task (MET) min per week. The following MET values were used to analyze different categories of IPAQ data activities: Walking = 3.3 METs, Moderate activity PA = 4.0, and METs and Vigorous PA = 8.0 METs. For all domains, minutes/week were converted to Metabolic Equivalent Task (MET)/minutes/week by multiplying total minutes/week by the MET values. All the domain-specific values were then added to get the total PA score. Adhering to the IPAQ scoring protocol, categorizing to low, moderate, and high physical activity levels was done [27]. Physical inactivity, which meant not engaging in adequate physical activity, was defined as physical activity < 600 MET min per week.

#### Anthropometric measurements

Height was measured using a microtoise steel tape and recorded to the nearest 0.5 cm. The subjects looked straight ahead with their head, back, and feet were touching the vertical support. Weight was measured without shoes on an electronic digital weighing scale to the nearest 100 g, and the scale was calibrated after each field session against the standard weights set. The BMI is calculated by weight in kilograms divided by the square of the height. The classification and cut-off points used were based on the anthropometry of adult Asians. A BMI of ≤18.49 kg/m2 was regarded as underweight, 18.50 kg/m2 to 23.00 kg/m2 as desirable, 23.01 kg/m2 to 27.50 kg/m2 as overweight and ≥ 27.51 kg/m2 as obese [28].

Statistical analyses were carried out using STATA data analysis and statistical software. Data are reported as the means ± SD and medians for continuous variables and proportions and percentages for categorical values. The characteristics of individuals with and without hypertension were compared using χ2 tests for categorical variables and independent t-tests for continuous variables. Statistically significant differences were determined using a two-sided *P* < 0.05. Pearson correlation coefficient was used to assed the correlation between commuting distance and total transport MET. Binary logistic regression was used to assess the associations between BP and physical inactivity with and without adjustments for potential confounders.

## Results

Of the 275 SOs invited for the study, 272 responded. Therefore, the response rate of SOs was 98.9%. Of the 760 MAs invited, 739 responded; hence, the response rate for MAs was 97.2%. Selected sociodemographic and occupational characteristics of SOs and MAs are given in Table 1. The mean ages were 44.1 (SD ± 9.0) and 42.1 (SD ±8.7) years for SOs and MAs, respectively. The majority were Sinhalese in both categories. A majority of the respondents among SOs (39.7%; *n* = 108) and MAs (43.3%; *n* = 320) were between 30 and 39 years.

**Table 1:** Sociodemographic/Economic and Occupational Characteristics of the Senior-Officers (*n* = 272) and Managerial Assistants (*n* = 739)

Variable	Senior Officers		Managerial Assistants	
	n	%	n	%
Age (years)				
30–39	108	39.7	320	43.3
40–49	73	26.8	228	30.8
50–60	91	33.5	191	25.9
Sex				
Female	156	57.4	578	77.3
Male	116	42.6	168	22.7
Level of education				
GCE Ordinary Level passed	0	0	17	2.3
GCE. Advanced level passed	9	3.3	100	13.5
Technical/diploma/vocational training	128	47.1	397	53.7
University degree	24	8.8	64	8.7
Postgraduate degree	111	40.8	161	21.8
Average monthly salary (Rs)				
10,000–29,000	135	49.6	712	96.3
30,000–49,000	107	39.4	27	3.7
≥ 50,000	30	11.0	0	0
Family income (Rs)				
≤ 20,000	4	1.5	32	4.3
21,000–59,000	160	58.8	612	82.8
60,000–99,000	80	29.4	72	9.8
≥ 100,000	28	10.3	23	3.1
*Occupational characteristics*				
Duration of service in the current workplace (years)				
≤ 5	178	65.4	457	61.8
6–10	64	23.5	214	29
11–20	19	7.1	40	5.4
≥ 21	11	4	28	3.8
Average work hours per week (hours)				
40	60	22.1	255	34.5
41–50	159	58.5	417	56.4
≥ 51	53	19.4	67	9.1
Commuting distance (km)				
≤ 10	67	24.6	200	27.1
11–20	106	39	191	25.8
21–30	39	14.3	113	15.3
31–40	19	7	70	9.5
≥ 41	41	15.1	165	22.3
Grade of work				
Special grade	58	21.3	22	3
Grade I	89	32.7	189	25.6
Grade II	56	20.6	305	41.3
Grade III	69	25.4	223	30.1

The main commuting method for SOs (59%) was private transport and MAs (64%) public transport. Thirty-nine-percent(*n* = 106) SOs and 25.8%(*n* = 191) MAs reported a commuting distance between 11 and 20 Km. The median distance from the current residence to the current workplace was 15 Km(range 0.25–130 Km) and 20 km(range of 0.2–131 Km) for SOs and MAs, respectively.

**Fig. 2:**
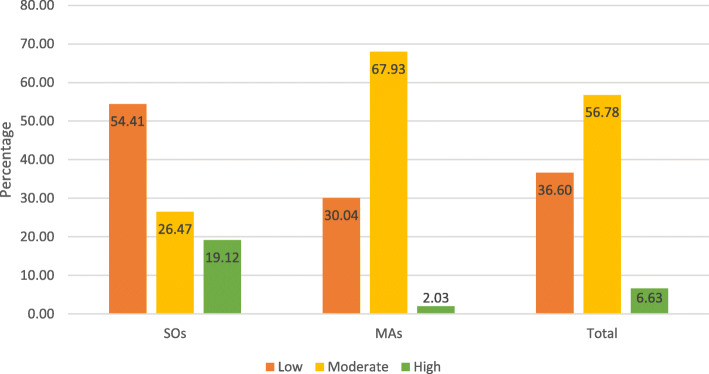
Levels of Physical activity in the study population

As shown in Fig. 2, socioeconomic variations in PA levels were observed as 54.4%(*n* = 148) senior-officers were involved in inadequate PA, whereas among the managerial assistants, only 30.0%(*n* = 222) were in the above category. Of the 1011 respondents, 37% (*n* = 370), 56.8%(*n* = 574), and 6.6%(*n* = 67) were involved in low, moderate, and high PA levels based on MET scores calculated using MET mins per week.

Considering the total physical activity levels, the mean MET/mins/week was 2739.5 (SEM 159.2) and 3343.9 (SEM128) for SOs and MAs, respectively (Table 2).

**Table 2:** Physical activity (in MET mins/day) stratified by sub-groups

Sub-group	Senior Officers		Managerial Assistants		p
	n	MET/ mins Mean (±SD)	n	MET/mins Mean (±SD)	
**Age-group (years)**					
30–39	107	2549.6 (±2276.8)	325	3131 (±3126.1)	0.039
40–49	73	2695 (±2640.2)	228	3947 (±4246.0)	0.003
50–60	91	2998.4 (±2968.5)	182	2970.4 (±2719.0)	0.94
**Sex**					
Male	113	2719.8 (±2562.5)	167	3597.8 (±2877.3)	0.0094
Female	158	2753.5 (±2669.7)	568	3269.4 (±3541.7)	0.08
**BMI categorization**					
Normal and underweight	85	2912.6 (±2910.3)	264	3771.6 (±3830.7)	0.031
Overweight	134	2681.4 (±2436.3)	314	3459.2 (±3623.6)	0.008
Obese	52	2606.1 (±2618.7)	157	3167.9 (±3592.6)	0.22
**MET based on activity**					
Work-related	271	296.4 (±798.4)	732	530.6 (±1416.4)	0.001
Transport related	272	464.4 (±748.2)	733	633.1 (±1223.1)	0.009
Leisure and sports-related	271	315.3 (±846.4)	735	241.2 (±732.5)	0.21
Domestic activity	272	1663.9 (±1746.6)	735	1924.5 (±2239.5)	<.001
Total Physical Activity	271	2739.5 (±2620.8)	735	3343.9 (±3445.7)	0.003

Among SOs and MAs, 68%(*n* = 69) and 59.9%(*n* = 132) of the hypertensives reported a commuting distance of ≤20 Km, respectively. Furthermore, the commuting distance was positively correlated(*p* < 0.05) with total transport MET among SOs and MAs.

The study revealed that the age- and sex-adjusted prevalence of hypertension was almost equal among SOs (32.9%; 95% CI = 27.4, 38.6) and MAs (33.01%; 95% CI = 29.6, 36.4) (*p* > 0.05) (Table 3). Of the diagnosed hypertensives, 47.5% (*n* = 145), were physically inactive/ less active and considering non-hypertensives, 34.7%(*n* = 245) were physically inactive/ less active.

**Table 3:** Prevalence of hypertension among the study population

Classification of hypertension	Senior officers *n* = 272		Managerial assistants *n* = 739	
	No.	%	No.	%
Normal	173	63.6	469	63.5
Pre-hypertension	11	4.0	53	7.2
Hypertension under control	24	8.8	49	6.6
Hypertension - Stage 1	58	21.3	147	19.9
Hypertension - Stage 2	4	1.5	7	1.0
Isolated systolic hypertension *	2	0.8	14	1.8
**Total**	**272**	**100.0**	**739**	**100.0**

*Systolic BP ≥140 mmHg and diastolic < 90 mmHg

Among the SOs diagnosed with hypertension, more than half (59.1%; *n* = 52) were physically inactive, while among MAs, 65.9% (*n* = 143) with hypertension were physically inactive. After adjusting for potential confounding factors being physically inactive was associated with a higher risk of hypertension among SOs [OR = 2.08 [95% CI; 1.07, 4.6] and MAs [OR = 2.8 [95% CI; 1.8, 4.6] (Table 4). After adjusting for potential confounding factors (age, sex, monthly income, education level, body mass index, and BMI), commuting distance of > 20 km was found to lower the odds of hypertension among SOs and MAs(OR = 0.713; 95% CI 0.4 to1.3; and OR = 0.63; 95% CI 0.46 to 0.87).

**Table 4:** Physical inactivity and hypertension among SOs and MAs

	Senior Officers			Managerial Assistants		
	Hypertensive N (%)	Unadjusted OR (95%CI)	Adjusted OR	Hypertensive N (%)	Unadjusted OR	Adjusted OR
Physical inactivity	52 (59.1)	2.21 (1.24–3.9)	2.08 (1.07–4.6)	143 (65.9)	2.8 (1.8–2.7)	1.33 (1.1–1.7)

Odds ratio (95% CI) for hypertensives were estimated using binary logistic regression analysis with controlling for the following; age > 40 years, male sex, Rs. > 100,000.00 per month monthly income, high body mass index, commuting distance <20Km per day and current alcohol consumption

## Discussion

This study mainly investigated the association between physical inactivity and hypertension and explored socioeconomic variations in physical activity in government officials in Sri Lanka. The interrelationship between physical inactivity and health is not a new concept. A crucial determinant of behavioral risk factors such as physical inactivity is adults’ socioeconomic status (SES), which is indicated by their educational attainment, income, and occupational category [29, 30]. The Whitehall Study involving a cohort 18,000 of male civil servants aged 20–64 years has reported that the social status of employees based on their seniority/ occupation affects morbidity and mortality in a wide range of diseases [31]. The current SES was proxied to the job category, which depicts the individual’s position in the social structure.

The study found that 54.4%(*n* = 148) SOs and 30.0%(*n* = 222) were physically inactive. Of the 1011 respondents, 37% (*n* = 370), 56.8%(*n* = 574), and 6.6%(*n* = 67) were involved in low, moderate, and high PA levels based on MET scores calculated using MET mins per week. It should be noted that reported physical inactivity levels are likely to underestimate the actual burden attributable to inactive lifestyles.

The current study showed domestic activity was the main mode, followed by transport-related activity among SOs and MAs. This finding is supported by another study conducted in Sri Lanka in which they reported that the main mode of energy expenditure is domestic work [32]. Domestic activities involve housework, household maintenance, yard work, and caring for the family. Increasing active travel leads to increase physical activity. The mode of traveling to work and the health benefits are not much researched, especially in Sri Lanka. Commuting to work is defined as regular travel between place of residence and work. Sedentary behaviour associated with sedentary commuting hinders physical activity and thereby leads to ill health. Commuting distance was positively correlated(*p* < 0.05) with total transport MET among SOs and MAs, meaning active travel was involved. These findings are supported by a cross-sectional study done in India [33]. Creating low-traffic neighborhoods (LTN) is also essential in this context to improve walking and cycling [34]. This study revealed that the mode of transportation depended on the SES. However, if LTN’s are implemented, this would increase active travel and the related public health benefits for all irrespective of their socioeconomic status.

Considering physical activity based on SES strata, it is evident that across all subgroups, there were significant differences between physical activity. Educational attainment and occupational category are indicators of an individual’s socioeconomic status, a key determinant of health, [35, 36] and help explain senior officers’ and managerial assistants’ physical activity patterns. The SOS and MAs differed in educational level and monthly family income (*p* < 0.05). The MAs reported lower physical inactivity (30%) than the SOs (54%). The mean MET/mins/week was 2739.5 (SEM 159.2) and 3343.9 (SEM128) for SOs and MAs, respectively, and the observed difference was significant (*p* = 0.003). These findings are consistent with those of other studies, showing variations in physical inactivity across socioeconomic status (SES) strata [37]. A study conducted in Sri Lanka which reports that high levels of education and older males have less physical activity supports the findings of the current study [17]. However, this study revealed that MAs, as opposed to SOs, reported significantly (*p* < 0.005) higher MET-based activity levels related to work, transport, and domestic activities. Senior officers travel in private vehicles, are more involved with meetings and paperwork, and work long working hours, leading to a sedentary lifestyle. The observed inverse relationship between age and physical inactivity is consistent with studies conducted elsewhere [38]. A possible explanation for this finding is that younger people opt for healthy habits and that with increasing age, employees become more sedentary.

Interestingly leisure and sports-related MET was higher among the SOs (*p* > 0.05). It could be that perceived lack of activity prompts the SOs to engage in sports-related activity when free. However, compared to other domains, leisure-time physical activity was low among both SOs (315 (SEM 846)) and MAs (241 (SEM 733)) MET/mins/week, and this is similar to studies done elsewhere [37, 39]. The Mexico City Diabetes Study, a cohort study, reported low levels of leisure-time physical activity levels (336 MET/ mins/ week) [39]. Despite the current knowledge of physical activity promoting an individual’s health, the practices among sedentary workers are obviously different. The rapid socioeconomic development is leading to a more sedentary type of lifestyle.

The present study carried out among administrators (SOs and MAs) confirms previously reported trends in the prevalence of hypertension and physical inactivity in Sri Lanka. The age and sex-adjusted prevalence of hypertension among 30–60 years SOs and MAs attached to the above offices were 32.9 per hundred population with a 95% CI of 27.4 to 38.6 and 33.01 per hundred population with a 95% CI of 29.6 to 36.4 respectively. The observed differences between the two percentages among SOs and MAs were not statistically significant (*p* > 0.05). Of the SOs 63.6% (*n* = 173) were normotensives and 4.0% (*n* = 11) were pre-hypertensives while 21.3% (*n* = 58), 1.5% (*n* = 4), and 0.7% (*n* = 2) were in the hypertension stage I, stage II and in the isolated systolic categories respectively. Considering MAs 63.5% (*n* = 469) were normotensives and 7.2% (*n* = 53) were pre-hypertensives, while 19.9% (*n* = 147), 1% (*n* = 7), and 1.9% (n = 14) were in the hypertension stage I, stage II and in the isolated systolic categories respectively. Of the hypertensive SOs and MAs, 52.3% (n = 46) and 35% (*n* = 76) were unaware they had hypertension, respectively. Of the patients diagnosed and on treatment for hypertension, 68.6% (*n* = 24) of SOs and 43% (*n* = 49) of MAs had controlled hypertension.

After adjusting for potential confounding factors (age, sex, monthly income, body mass index, commuting distance, high BMI, and alcohol intake), physically inactive participants had a greater odds of having hypertension than the physically active participants. This is similar to the findings from a cohort study involving 2282 participants, who were followed for 20 years, which reported accumulating < 1 MET/min/week of occupational moderate to vigorous physical activity was associated with a 47% higher risk of hypertension (HR 1.47, CI 95% 1.13, 1.90) and accumulating < 1 MET/min/week of leisure moderate to vigorous physical activity was associated with 29% higher risk of hypertension 1.29 (1.01, 1.66). Physical activity reduces the risk of hypertension by reducing body weight, reducing psychological stress, improving insulin sensitivity, and reducing sympathetic activity [40]. There can be several plausible pathways through which commuting distance lowers the odds of hypertension. In countries like Sri Lanka, long-distance commuting may involve active transport. Studies elsewhere have reported that active commuting was associated with lower BMI, especially if the commuting distance is> 20 km [41, 42]. Although the study showed that a longer commuting distance lowered the odds of hypertension, it was not a significant association (OR = 0.713; 95% CI 0.4 to1.3). This may be due to the small sample size of SOs (*n* = 272) compared to MAs (*n* = 739) and because most of the SOs used a private vehicle for transportation. Considering the total transport MET, a positive correlation of total transport MET, and the association between long-distance travel and hypertension, our results support promoting active transportation methods for employees.

The study is not without limitations. As this study was conducted in the government administration offices in the Colombo district, the results may not apply to all administrative employees in the country, which is considered a limitation. Physical activity was based on self-reports which required a 7-day recall. This might not necessarily reflect accurate physical activity levels across lifespan. Recall bias and reporting socially desirable practice may have played a role in bias towards null.

Additionally, although we controlled for all the known confounders, the residual confounders may exist. Finally, the study identified physical inactivity as a correlate of hypertension through a cross-sectional comparative study design. This precluded the assessment of the temporal relationship between hypertension and inactivity.

## Conclusions and recommendations

Our findings suggest that physical inactivity in employees is alarmingly high. Physical inactivity was prevalent among the hypertensive individuals compared to non-hypertensive participants. Physical inactivity is an important independent contributor to hypertension among employees. The present study adds to the evidence of wider influencing factors related to physical inactivity as socioeconomic variations in PA levels existed. The socioeconomic variations observed in physical activity levels highlight the importance of considering social differences to ensure the successful and equitable implementation of programs to prevent hypertension. Higher commuting distance is positively correlated with total transport MET, and long commuting distance (> 20 km) lowered the odds of hypertension among SOs and MAs. This may imply that active travel increases physical activity and thereby results in lowering the odds of hypertension. Further studies are necessary to explore the association of hypertension, physical inactivity, and commuting distance. Policies and environment to promote and at least the recommended amounts of physical activity among employees necessary to avert premature deaths due to chronic illnesses such as hypertension should be prioritized. Screening and diagnosis need to be coupled with treatment to achieve good control of diseases such as hypertension. Implement well-integrated worksite health promotion activities, which include regular periodic screening, surveillance, and provision of healthy opportunities at the workplace like a healthy diet, physical activity, mental health & well-being, coupled with policy commitment. The actions should be continued with inbuilt monitoring and evaluation.

## Data Availability

The datasets generated and/or analysed during the current study are not publicly available due to limitations of ethical approval involving the patient data and anonymity but are available from the corresponding author on reasonable request.

## Abbreviations

BMI: Body Mass Index
CI: Confidence Interval
IPAQ: International Physical Activity Questionnaire
MAs: Managerial Assistants
MET: Metabolic-Equivalent-of-Task
OR: Odds Ratio
PA: Physical activity
SES: Socio-economic status
SOS: Senior officers
SD: Standard Deviation
WHO: World Health Organization
